# Recreational walking and the perceived local environment among socioeconomic disadvantaged adults: Suzannah D'Hooghe

**DOI:** 10.1093/eurpub/ckac130.164

**Published:** 2022-10-25

**Authors:** S D'Hooghe, B Deforche, D van Dyck, K De Ridder, S Vandevijvere, N van de Weghe, S Dury

**Affiliations:** 1 Department of Epidemiology and Public Health, Sciensano, Elsene, Belgium; 2 Department of Public Health and Primary Care, Ghent University, Ghent, Belgium; 3 Department of Movement and Sport Sciences, Ghent University, Ghent, Belgium; 4 Department of Geography, Ghent University, Ghent, Belgium; 5 Department of Educational Sciences, Vrije Universiteit Brussel, Brussels, Belgium; 6 Department of Movement and Sport Sciences, Vrije Universiteit Brussel, Brussels, Belgium

## Abstract

**Background:**

Insufficient physical activity (PA) is a risk factor for obesity and non-communicable diseases and seems more prevalent among socioeconomically disadvantaged (SED) adults. Recreational walking (RW) is an important type of leisure time PA which can be done for free and without specific equipment or facilities. Environmental factors influencing PA may be particularly important for SED adults who are more reliant on their direct environment. The objectively measured environment is not always congruent with the perception of residents, and this may differ by socioeconomic group. This study aims to identify the local environmental factors important for RW as perceived by SED adults.

**Methods:**

This study is part of the CIVISANO project. Purposeful convenience sampling was used to recruit 38 SED adults (25-65 y/o) in two Flemish semi-urban municipalities. Individual walk-along interviews have been performed in the participants’ neighborhood. 20 participants joined the focus group discussion (n = 4) that were intended for member checking, to categorize identified environmental factors and to identify local actions to promote recreational walking. MaxQDA was used for content analysis.

**Results:**

The way the environment is perceived by SED adults plays an important role in their RW behavior. Results demonstrate the interrelation of different environmental types (physical, sociocultural, economic, political and information) and sizes (micro, meso, macro). Improving communication and knowledge transfer, stimulation of physical, sociocultural, and economic accessibility, and promotion of physical and social safety are identified most important action points for the local government to facilitate RW.

**Conclusions:**

Our findings indicate that the perceived local environment can play an important role in promoting RW among SED adults. Future studies should investigate if changes in these environments and in residents’ perception lead to changes in RW behavior of SED adults.

**Key messages:**

- The way the environment is perceived by socioeconomic disadvantaged adults plays an important role in their recreational walking behavior.
- Participation of community members allows to integrate novel and community-sourced ideas for practical intervention planning with high external validity.

